# Novel Genetic Variants Associated with Child Refractory Esophageal Stricture with Food Allergy by Exome Sequencing

**DOI:** 10.3390/nu9040390

**Published:** 2017-04-15

**Authors:** Min Yang, Min Xiong, Huan Chen, Lanlan Geng, Peiyu Chen, Jing Xie, Shui Qing Ye, Ding-You Li, Sitang Gong

**Affiliations:** 1 Department of Gastroenterology, Guangzhou Women and Children’s Medical Center, Guangzhou Medical University, 9 Jinsui Road, Guangzhou 510623, China; mandy1005@163.com (H.C.); genglan_2001@hotmail.com (L.G.); chenpei.y@163.com (P.C.); xiejing616@126.com (J.X.); 2 Division of Experimental and Translational Genetics, Children’s Mercy Hospital, University of Missouri Kansas City School of Medicine, 2401 Gillham Road, Kansas City, MO 64108, USA; mxiong2@cmh.edu (M.X.); sqye@cmh.edu (S.Q.Y.); 3 Division of Gastroenterology, Children’s Mercy Hospital, University of Missouri Kansas City School of Medicine, 2401 Gillham Road, Kansas City, MO 64108, USA

**Keywords:** children, exome-seq, food allergy, genetic variants, refractory esophageal stricture

## Abstract

Background: Refractory esophageal stricture (RES) may be attributed to food allergy. Its etiology and pathogenesis are not fully understood. Identification of novel genetic variants associated with this disease by exome sequencing (exome-seq) may provide new mechanistic insights and new therapeutic targets. Methods: To identify new and novel disease-associating variants, whole-exome sequencing was performed on an Illumina NGS platform in three children with RES as well as food allergy. Results: A total of 91,024 variants were identified. By filtering out ‘normal variants’ against those of the 1000 Genomes Project, we identified 12,741 remaining variants which are potentially associated with RES plus food allergy. Among these variants, there are 11,539 single nucleotide polymorphisms (SNPs), 627 deletions, 551 insertions and 24 mixture variants. These variants are located in 1370 genes. They are enriched in biological processes or pathways such as cell adhesion, digestion, receptor metabolic process, bile acid transport and the neurological system. By the PubMatrix analysis, 50 out of the top 100 genes, which contain most variants, have not been previously associated with any of the 17 allergy-associated diseases. These 50 genes represent newly identified allergy-associated genes. Those variants of 627 deletions and 551 insertions have also not been reported before in RES with food allergy. Conclusions: Exome-seq is potentially a powerful tool to identify potential new biomarkers for RES with food allergy. This study has identified a number of novel genetic variants, opening new avenues of research in RES plus food allergy. Additional validation in larger and different patient populations and further exploration of the underlying molecular mechanisms are warranted.

## 1. Introduction

Benign esophageal stricture in children may be caused by caustic ingestion, peptic ulcer disease, congenital anomaly and esophageal surgery [1]. Endoscopic balloon dilatation is safe and effective in the majority of children with esophageal stricture [2]. Refractory esophageal stricture (RES) refers to those that do not respond to repeated dilations, which may be attributed to food allergy. There have been reports that cow’s milk protein allergy may develop after gastrointestinal surgeries or injuries, possibly due to a disruption of the mucosal barrier [3,4,5]. We speculated that genetic susceptibility contributes to the development of food allergy in children with RES. The aim of this study was to use whole-exome sequencing to identify potential new underlying genetic variants in those children.

## 2. Methods

### 2.1. Patients

The institutional ethics committee of Guangzhou Women and Children’s Medical Center approved this study protocol (20170301).

We included three children diagnosed with RES and multiple food allergies (Table 1). Patients’ clinical features, laboratory tests and treatment outcomes were reviewed. After initial clinical evaluation, patients underwent endoscopic examination to assess for esophageal stricture. Subsequent endoscopic balloon dilatation was performed according to the standard protocol. Balloon dilatation was repeated based upon the patient’s symptom of dysphagia. All three patients were considered refractory to esophageal balloon dilatations and were found to have a positive serum IgE (sIgE) response to milk, eggs and peanuts. After treatment with dietary allergen exclusion, all patients showed clinical improvement and resolution of esophageal stricture.

### 2.2. Exome Capture and Illumina Sequencing

Genomic DNA from each patient’s blood was isolated using DNeasy Blood & Tissue Kit (Cat. No. 69504, Qiagen, Valencia, CA, USA) following the manufacturer’s protocol. The libraries for exome sequencing were created using the Nextera Rapid Capture Exome and Expanded Exome Kit (Illumina, San Diego, CA, USA). TruSeq SBS v3 whole-exome, 2 × 101 paired end sequencing was performed on the Illumina Hiseq1500 platform according to the manufacturer’s instructions.

### 2.3. Read Mapping and Variant Calling

The paired-end reads were aligned to the hg19 human reference genome using BWA (Burrows-Wheeler Aligner) [6]. Picard Sort Sam was used to convert the SAM (sequence alignment map) file into a BAM (binary sequence alignment map) file, and to sort the BAM file order by starting positions [7]. Picard Mark Duplicates was used to remove PCR duplicates (7). The read group information was very important for downstream GATK (genome analysis toolkit) functionality. Picard Add Or Replace Read Groups replaced all read groups in the input file with a single new read group and assigned all reads to this read group in the output BAM [7]. Samtools indexed the BAM file for fast random access to the human reference genome [8]. The Genome Analysis Toolkit (GATK) pipeline was used for recalibration, local realignment around indels and variation calling [9]. SNPs with a quality score less than 20, a depth of coverage less than 4, call ratios below 0.85 and HWE (Hardy-Weinberg equilibrium) below 10^−6^ were removed. SNP & Variation Suite™ software (Golden Helix, Inc., Bozeman, MT, USA) was used for variation annotation and genotype association tests. Predictions of protein functional effect changes with those variants were performed by Sift and Polyphen 2 [10,11]. DAVID was utilized to perform Gene Ontology to identify the cellular biological processes of genes with pediatric refractory esophageal stricture with food allergy SNPs [12]. PubMatrix analysis [13], a multiplex literature mining tool, was used as described previously [14] to build the relationship between genes with milk-protein allergy variations and allergy-associated diseases in PubMed.

## 3. Results

**Figure 1:**
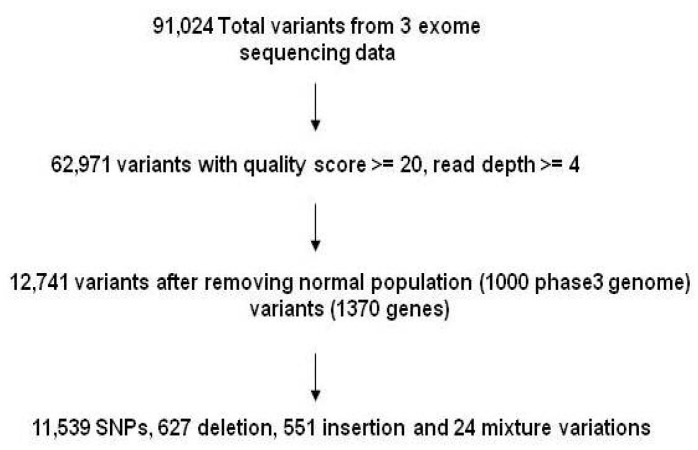
Workflow of identifying new genetic variants in three children with RES plus food allergy. Identified total variants were first subjected to the quality control and then filtered against ‘normal variants’ of the 1000 Genomes Project exome sequences (quality score ≥ 20, read depth ≥ 4). The remaining variants were applied for the further analyses.

**Figure 2:**
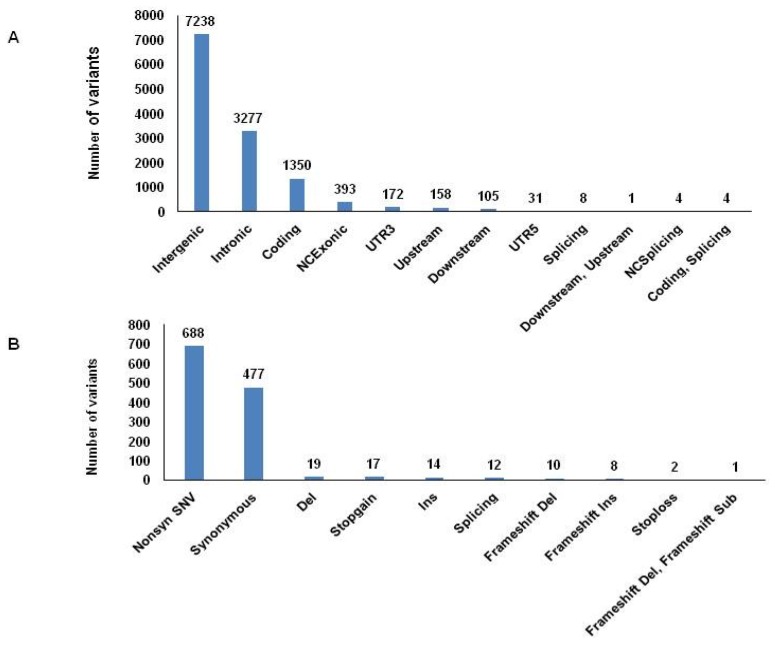
Variant location and type analyses. (**A**) Variant locations. Variant distribution in each of 12 regions are displayed in bar graphs. UTR, untranslated region; NC, noncoding region. (**B**) Variant types. Distribution of 10 different variant types are presented in bar graphs. Del, deletion; Sub, substitution.

For the discovery phase, 91,024 variants were detected by whole-exome sequencing in three patients. After filtering lower quality alleles, 62,971 high quality variants were kept for the subsequent analysis. 12,741 variants of 1370 genes were kept after filtering out ‘healthy’ wildtype or SNPs from the 1000 genome phase 3 population [15] found in the three patients, and 90.57% of the variants were SNPs (Figure 1). The majority of variants were located in non-coding intronic and intergenic regions (82.53%) (Figure 2A). Further analysis of variants within the coding regions revealed that 55.13% of variants identified in the three patients were nonsynonymous SNPs (Figure 2B). Interestingly, phenotypic damage analysis of these variants by SIFT and Polyphen 2 HVAR showed that 204 and 89 variants existing in the three patients were predicted as damaging variants, respectively.

**Figure 3:**
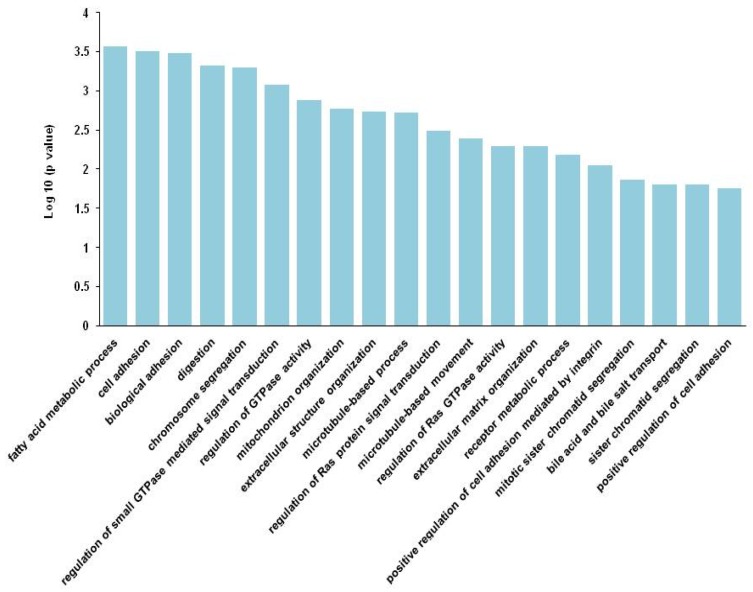
Top 20 biological processes’ enrichment of genes with variants found in all three patients. All disease-associated genes with variants found in all three patients were supplied for biological process analysis using the software program DAVID, as described in Methods. Only the top 20 biological processes are presented.

**Figure 4:**
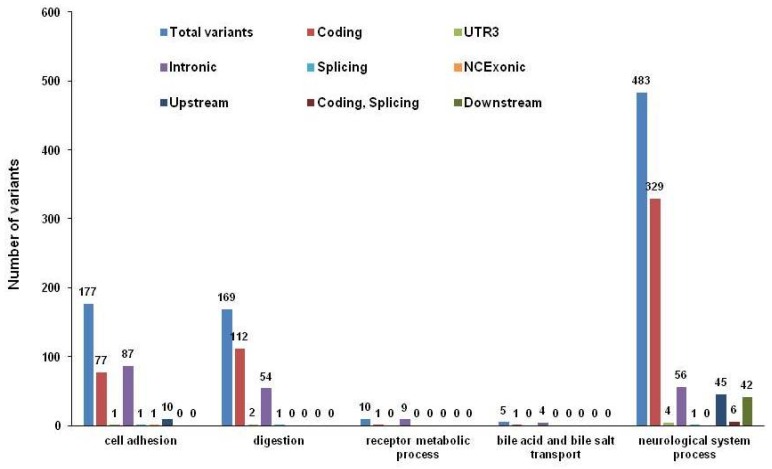
Variant type distribution in five relevant pathways or biological processes. Variant type (eight) distributions in five relevant pathways or biological processes are displayed in bar graphs.

The 12,741 variants identified in the three patients were located in 1370 genes. Among them, 182 genes were enzymes, 48 genes were transporters and 27 genes were ion channels. The 30 significant biological processes were enriched for genes with these variants (*p* < 0.05). Figure 3 shows the top 20 biological processes’ enrichment. Sixty-two genes with 177 variants were enriched in cell adhesion, 15 genes with 169 variants were enriched in digestion, seven genes with 10 variants were enriched in receptor metabolic process, four genes with five variants were enriched in bile acid and bile salt transport, and 82 genes with 483 variants were enriched in neurological system process, all of which are important biological processes for pediatric refractory esophageal stricture combined with food allergy (Figure 4). The majority of these variants in digestion (66.27%) and neurological system process (68.12%) were located in coding regions (Figure 4).

**Figure 5:**
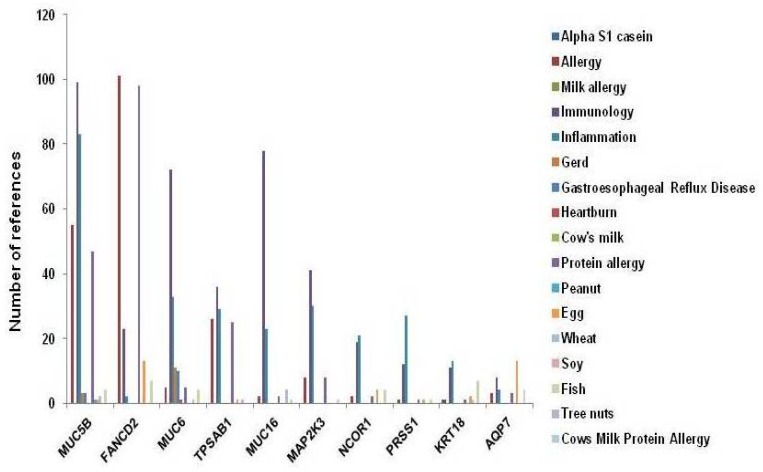
Top 10 PubMatrix genes with variants in splicing and coding regions found in all three patients. Top genes were applied to PubMatrix analysis to probe whether they have been previously associated with any of the 17 allergy-related diseases or conditions. The top 10 genes with the most previous association reports in literature are presented.

To identify potential etiologic genes, we ranked variant counts by genes. Three hundred and fifty-six genes had splicing and coding region variations, and the genes MUC6, OR8U1, OR8U8, PDE4DIP and KCNJ12 ranked in the top five. To further mine relationships between genes with RES with food allergy variations and allergy-associated diseases in PubMed, we submit the top 100 genes and 17 allergy-associated diseases into PubMatrix. This approach identified the genes MUC5B, FANCD2, MUC6, TPSAB1, MUC16, MAP2K3, NCOR1, PRSS1 and KRT18 as being linked to the most allergy terms (Figure 5).

## 4. Discussion

We identified three children with refractory esophageal stricture who were found to have food allergies to milk, eggs and peanuts. One patient had an esophageal mucosal eosinophil count of 35/hpf, and most likely has eosinophilic esophagitis. Two other patients had esophageal mucosal eosinophil counts of 9/hpf and 12/hpf, respectively, and did not meet histological criteria (≥15 eos/hpf) for eosinophilic esophagitis [16]. All patients responded well to dietary allergen exclusion, with resolution of esophageal strictures. To our knowledge, this is the first report associating food allergies with complicated refractory esophageal stricture in children who subsequently responded well to dietary allergen exclusion. Based on our report, it is important to consider cow’s milk protein allergy or multiple food allergies in children with refractory esophageal stricture.

This study applied whole-exome sequencing analysis to systematically identify genome-wide coding variants which may underlie genetic susceptibility to pediatric refractory esophageal stricture with food allergy from three children. Our analyses identified 12,741 variants of 1370 genes in these patients after filtering the 1000 Genome Project Phase 3 controls [15]. Three hundred and fifty-six genes with 1350 variants had splicing and coding region variations, among them 688 variants were nonsynonymous SNPs. The potential candidate genes in pediatric refractory esophageal stricture with food allergy are involved in ion channel, transporter, cell adhesion, digestion, receptor metabolic process, bile acid and bile salt transport and neurological system processes. These biological functions and pathways have a major impact on esophageal function, which is consistent with the pathogenesis of pediatric refractory esophageal stricture with food allergy.

Through searching the literature for the top 100 found genes in the three patients with refractory esophageal stricture with food allergy variations, 50 genes were previously linked to 17 allergy-associated diseases, 21 genes were previously associated with allergy, and 19 genes were linked specifically to protein allergy. This finding lends a strong support that WES (whole exome sequencing) is a promising approach to identify genetically susceptible genes linked with both refractory esophageal stricture and food allergy. However, none of these top genes are linked to cow’s milk and milk allergy, and 50 genes have not been previously associated with any of the 17 allergy-associated diseases. These 50 genes represent newly identified allergy-associated genes, opening new avenues to investigate new genetic risk factors in refractory esophageal stricture combined with food allergy.

Among top candidate genes, the MUC6 gene encodes a secreted glycoprotein that plays an important role in the cytoprotection of epithelial surfaces and mechanical trauma in the gastrointestinal tract [17,18]. Eighty-six variations were found in MUC6 splicing and coding regions in the three patients with RES and food allergies. Ten gastroesophageal reflux diseases and five protein allergy studies in the literature were reported to link to MUC6.

The majority of SNPs (82.53%) identified in this study map to non-coding regions of the genome, which complicates the analysis of these variations. However, this is a common finding in GWAS (genome-wide association study) studies and suggests that non-coding SNPs are located in functional regulatory regions, such as splicing regulatory elements, enhancer elements, DNase hypersensitivity regions and chromatin marks [19]. Thus, these non-coding SNPs may regulate the expression of nearby genes.

## 5. Conclusions

This study has demonstrated the power of WES to identify new genetic risk factors within the whole-exome scale in children with refractory esophageal stricture as well as food allergies. It should be mentioned that our study is limited to only three patients, and thus no solid conclusion can be drawn without a proper control and a large sample size. Replication of our findings in larger and different populations, in addition to experimental delineation of the underlying molecular mechanisms, is warranted to validate the candidate variants identified here as true genetic biomarkers and to develop them into potential therapeutic targets in refractory esophageal stricture presenting with food allergy.

## Figures and Tables

**Table 1:** Characteristics of children with refractory esophageal strictures. EBD: esophageal balloon dilatation; sIgE: serum IgE.

Case	1	2	3
Age (years)	12	2.5	4
Sex	male	male	male
Age at stricture diagnosis	8 years	1 month	2 years
Cause	Caustic agent	Post-surgery (esophageal atresia)	Post-surgery (Hiatal hernia)
Initial stenosis diameter (mm)	1	5	<1
EBD (times)	18	7	6
Esophageal mucosal eosinophil/hpf	9	35	12
sIgE			
Milk	+	+	+
Egg	+	+	+
Wheat	+	-	+
Peanut	+	+	+
Soy	-	-	+
Fish	-	-	+
Others	rice	crab	rice
Treatment	Dietary allergen exclusion	Elemental formula	Dietary allergen exclusion
Follow-up duration (months)	14	12	26
Resolution of esophageal stricture	Yes	Yes	Yes
